# Association Between Calcaneal Inclination Angle and Spinal and Lower Limb Alignment: A Retrospective Radiographic Analysis

**DOI:** 10.3390/diagnostics16060921

**Published:** 2026-03-19

**Authors:** Yunhee Han, Seojae Jeon, Hyeonjun Woo, Wonbae Ha, Tae-Yong Park, Jin-Hyun Lee, Junghan Lee

**Affiliations:** 1 Department of Korean Medicine Rehabilitation, College of Korean Medicine, Wonkwang University, 460 Iksan-daero, Iksan-si 54538, Republic of Korea; kmedhyh@gmail.com (Y.H.); ha0530@gmail.com (W.H.); 2 Korea Institute of Integrative Medicine, 121 Rohaseu-ro, Anyang-myeon, Jangheung-gun 59338, Republic of Korea; fr1771@naver.com (S.J.); woohyeonjun@gmail.com (H.W.); 3 Catholic Kwandong University International St. Mary’s Hospital, 25 Simgok-ro 100 Beon-gil, Seo-gu, Incheon 22711, Republic of Korea; parktae9822@gmail.com (T.-Y.P.); doolyjinhyun@empal.com (J.-H.L.)

**Keywords:** foot, pes planus, pes cavus, foot deformities, posture, spinal alignment, pelvic alignment, lower extremity, manual therapy, retrospective studies

## Abstract

**Background/Objectives:** This study aimed to clinically investigate how variations in foot morphology influence spinal and lower limb alignment, based on the concept of an ascending kinetic chain. **Methods:** We analyzed the medical records of 100 patients who met the inclusion criteria. The X-ray image data used in the analysis included weight-bearing lateral views of both feet, whole-spine anteroposterior (AP) and lateral views, and full-length standing AP scanograms of the lower legs. In the obtained X-ray images, Calcaneal Inclination Angle (CIA), Tibiotalar Tilt Angle (TTA), Tibiotalar Angle (TA), Quadriceps Angle (Q-angle), Pelvic Incidence (PI), Pelvic Tilt (PT), Sacral Slope (SS), and L1–S1 Lordosis (LL) were measured. Participants were categorized into subgroups based on their CIA values: Pes Planus, Normal, and Pes Cavus. These subgroups were analyzed by foot orientation (right and left) using one-way analysis of variance (ANOVA) and Pearson correlation coefficient analysis. **Results:** The one-way ANOVA identified significant differences in mean right foot PT values among subgroups. Correlation analysis shows moderate associations between foot CIA and Q-angle of the knee, as well as pelvic parameters including PI, PT, SS, and LL. **Conclusions:** Analysis of the correlation between foot parameters and body alignment, in the context of diagnostic and evaluative aspects of Chuna manual medicine (CMM), revealed moderate correlations among the foot, ankle, knee, pelvis, and lumbosacral regions. These findings suggest that foot morphology may play a clinically relevant role in posture-related disorders and could contribute to preventive and corrective strategies for musculoskeletal alignment.

## 1. Introduction

The foot, the most distal component of the lower-limb kinetic chain, maintains balance while providing a relatively small support base. Even minor changes in this area can interfere with the postural control strategy [1]. Therefore, structural abnormalities of the foot can affect the entire motion chain, and when combined with balance disorders, proprioception issues, and changes in the proximal joints, may cause functional instability [2]. In this context, pes planus and pes cavus are conditions characterized by congenital or acquired changes related to midfoot height, which can cause musculoskeletal problems, including those in the knee and pelvis, and may also affect the spinal joints, leading to acute and chronic pain as well as structural changes in body alignment [3].

The structure and form of the foot are generally diagnosed based on the patient’s history, physical examination, and weight-bearing radiographs of the foot and ankle [4]. Among these, lateral weight-bearing radiographs are considered the standard method for diagnosing and evaluating adult foot deformities [5].

Recent research trends related to CMM diagnosis include studies using radiographic imaging, such as reliability studies comparing palpation with plain radiography for lumbar diagnosis [6], and systematic reviews investigating plain radiography and manual examination methods used in pelvic displacement diagnosis [7]. However, research related to feet remains limited. Regarding the effects of foot hyperpronation on various body parts, one study revealed a relationship between foot hyperpronation and pelvic internal rotation using a motion analysis system [8]. Another study investigated the effect of foot hyperpronation on spinal alignment in the standing posture using a motion analysis system [9]. However, research on the overall influence of the foot structure and form on body alignment, including the ankle, knee, pelvis, and lumbosacral regions, has not been reported.

Chuna manual medicine (CMM) is an academic discipline that studies the comprehensive processes of diagnosis, treatment, and evaluation of Chuna manual therapy as performed by Korean medicine doctors. When evaluating posture and body alignment from a holistic perspective in CMM, they are generally divided into ascending, descending, and pelvic girdle patterns. Among these, the ascending pattern refers to a misalignment of the lower body, such as foot hyperpronation, that leads to misalignment of the upper body; the foot plays an important role in this regard [10].

Previous studies examining the relationship between foot posture and proximal alignment have primarily relied on motion analysis systems or clinical observational approaches. However, radiographic investigations simultaneously evaluating foot morphology and spinopelvic parameters across multiple anatomical regions remain limited. Therefore, the present study provides a radiographic perspective on the ascending kinetic chain by analyzing correlations between foot morphology and ankle, knee, pelvic, and lumbosacral alignment parameters using standardized weight-bearing radiographs.

Therefore, this study aimed to analyze the correlation between foot, ankle, knee, pelvic, and lumbosacral alignments using plain radiographs in healthy adult men and women, with a focus on foot parameters. Based on this, we aimed to establish evidence for body alignment evaluation centered on the foot by applying CMM’s holistic diagnostic and therapeutic approach to CMM.

## 2. Materials and Methods

### 2.1. Selection of Research Subjects

This study was conducted retrospectively through a review of patients’ medical records. The study targeted patients who met the pre-established inclusion and exclusion criteria and had received treatment at Wonkwang University Korean Medicine Hospital between March 2021 and June 2023. The radiographs were originally obtained as part of routine clinical evaluations for musculoskeletal complaints such as lower back pain, postural imbalance, or lower-limb discomfort.

The sample size was determined based on a priori power analysis using G*Power 3.1 software to ensure adequate statistical power (0.80) at a significance level of 0.05 and a medium effect size (f = 0.25) for ANOVA tests. The required minimum sample size was calculated to be 34 participants per group. Because of the relatively small number of participants classified as pes planus, subgroup analyses were considered exploratory and were interpreted with caution. These analyses were conducted primarily to identify potential biomechanical trends rather than to draw definitive statistical conclusions. Although an a priori sample size estimation using G*Power was conducted for the overall cohort, the subgroup analyses based on foot morphology were exploratory in nature and were not intended for confirmatory statistical inference.

Prior to the study, approval was obtained from the Institutional Review Board of Wonkwang University Korean Medicine Hospital (IRB No. WKUIOMH-IRB-2021-08).

#### 2.1.1. Inclusion Criteria

(a) Adult men and women aged 20–60 years.
(b) Patients who underwent weight-bearing lateral radiographs of both feet, whole-spine anteroposterior (AP) and lateral views, and full-length standing AP scanograms of the lower legs.

#### 2.1.2. Exclusion Criteria

(a) History of trauma or surgery that could cause structural problems in the lumbar spine (e.g., fracture, osteoporosis, and lumbar surgery).
(b) History of diseases causing deformity of the lumbar structure (e.g., congenital malformation of the lumbar spine, ankylosing spondylitis, idiopathic scoliosis, tumors, and spina bifida).
(c) Patients deemed unsuitable for participation in this study by the principal investigator, including those with (1) severe musculoskeletal pain that limited standing or walking posture during imaging, (2) poor radiographic image quality due to motion artifacts or improper positioning, or (3) any comorbid neurological or systemic disease that could affect spinal or lower-limb alignment.

### 2.2. Study Design

Based on the patient’s medical records, the following variables were collected: sex, age, blood pressure, pulse rate, body temperature, height, weight, body mass index, and X-ray imaging data (weight-bearing lateral radiographs of both feet, whole-spine AP and lateral views, and full-length standing AP scanograms of the lower legs). For personal information protection, all imaging data were anonymized before use.

In this study, to evaluate foot structure and form, the calcaneal inclination angle (CIA) was measured on lateral weight-bearing radiographs of the foot [6]. Additionally, to identify body misalignment caused by lower body deformities such as pes planus and pes cavus, measurable parameters of the ankle, knee, pelvis, and lumbosacral region were selected from previous studies using plain radiographs. Correlation analysis was performed between foot parameters and ankle, knee, pelvic, and lumbosacral alignment parameters in groups divided according to foot structure based on CIA measurements. Although full-length scanograms were available, limb length discrepancy was not specifically evaluated because the primary objective of the present study was to examine angular alignment relationships between the foot and spinopelvic structures.

### 2.3. Body Alignment Parameters

#### 2.3.1. CIA

**Figure 1:**
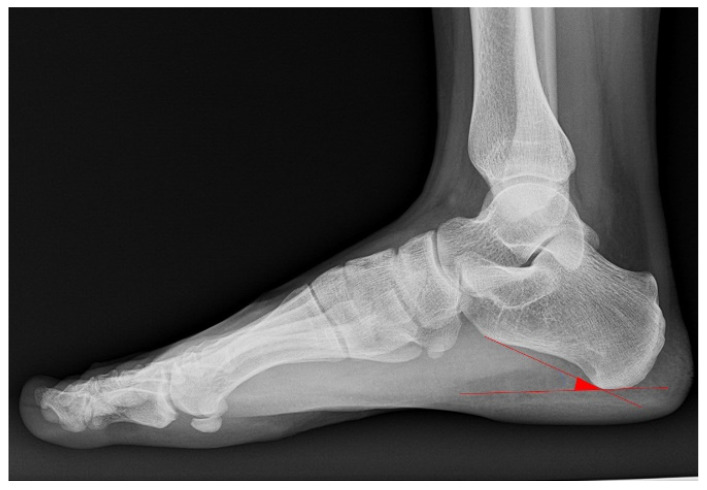
Illustration of foot angular parameter definition. The red lines indicate the calcaneal pitch angle.

The angle between the plantar calcaneal surface line and the horizontal plane was measured using weight-bearing lateral foot radiographs. For the foot CIA, 17° to less than 25° was considered normal; values less than 17° were classified as pes planus [11], and values greater than 25° were classified as pes cavus [12,13,14] (Figure 1). Generally, when the CIA is within the normal range, the heel height is normal, and the foot arch is stably supported. However, a decreased CIA indicates pes planus, while an increased CIA indicates pes cavus, both of which can affect leg and knee alignments.

#### 2.3.2. Alignment Parameter of the Ankle

**Figure 2:**
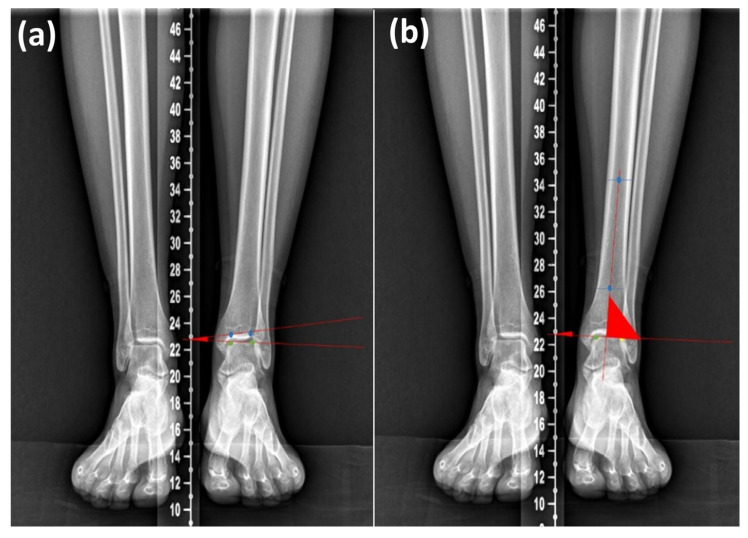
Illustration of (**a**) tibiotalar tilt angle and (**b**) tibiotalar angle. Green dots indicate the distal tibial articular surface (tibial plane), and blue dots indicate the talar dome in (**a**) and the tibial axis in (**b**). The red lines represent the reference lines used to measure each angle.

The tibiotalar tilt angle (TTA) and tibiotalar angle (TA) were measured on weight-bearing AP radiographs of the lower extremities. The TTA was measured as the angle between the anatomical axis of the tibia and the tangent line to the superior surface of the talar dome. The TA was measured as the angle between the anatomical axis of the tibia and the tibial longitudinal axis (Figure 2). The TTA and TA are parameters used to evaluate ankle and hindfoot alignment and to assess ankle instability [15].

#### 2.3.3. Alignment Parameter of the Knee

**Figure 3:**
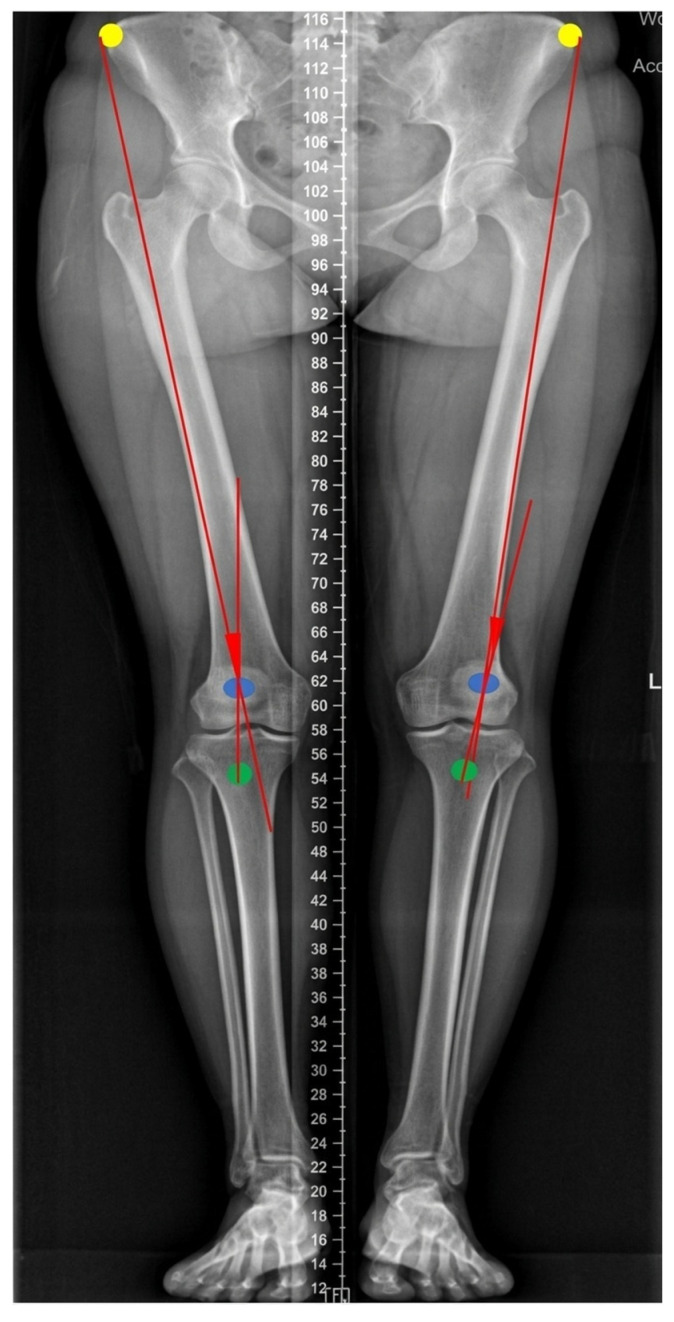
Illustration of Q-angle parameter definition. Yellow dots indicate the anterior superior iliac spines; blue dots indicate the center of the patella; and green dots indicate the tibial tubercles. The red lines indicate the Q-angle.

For the knee, the quadriceps angle (Q-angle) was measured on weight-bearing AP radiographs of the lower extremities. The Q-angle was defined as the angle formed between an imaginary line connecting the anterior superior iliac spine and the midpoint of the patella, and the line connecting the tibial tubercle to the center of the patella projected proximally (Figure 3) [16]. The Q-angle is an anatomical indicator of femoral and tibial alignment [17], and represents the frontal plane resultant force vector of the quadriceps and patellar tendon acting on the patella [18].

#### 2.3.4. Alignment Parameter of Pelvis

**Figure 4:**
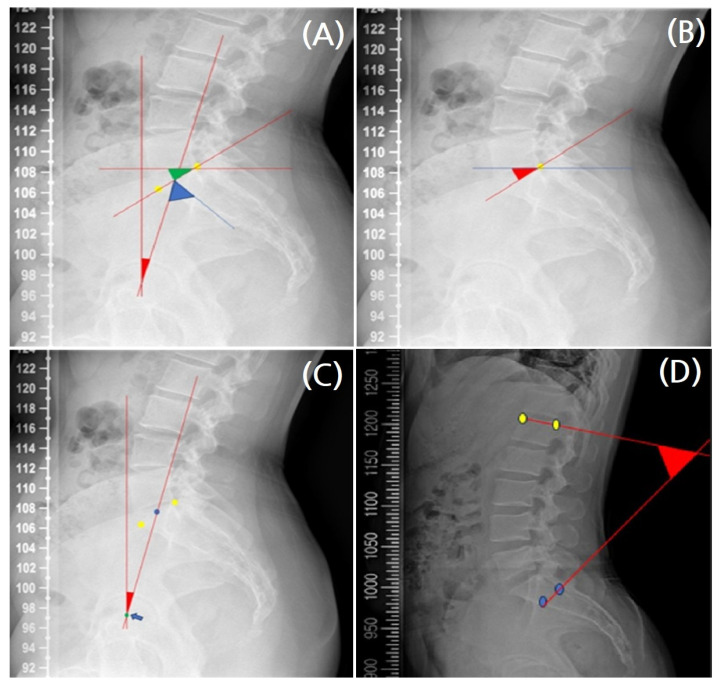
Illustration of (**A**) pelvic incidence, (**B**) sacral slope, (**C**) pelvic tilt, and (**D**) L1-S1 lordosis parameter definition. (**A**) Yellow dots: femoral head centers; Red arrowhead: pelvic tilt angle; green arrowhead: sacral slope angle; blue arrowhead: pelvic incidence angle. (**B**) Yellow dot: top of S1; red arrowhead, sacral slope angle. (**C**) Yellow dots: left and right upper margins of S1; blue dot: center of the upper margin of S1; red arrowhead: angle of pelvic tilt; blue arrow: femoral head axis. (**D**) Yellow dots: top left and top right of L1; blue dots: bottom left and bottom right of S1 vertebral body; red arrowhead: Cobb’s angle between L1 and S1. The red lines indicate the reference lines used for each parameter measurement.

For the pelvis, four parameters were measured on weight-bearing whole-spine lateral and AP radiographs. First, the pelvic incidence (PI), pelvic tilt (PT), and sacral slope (SS) were measured using lateral whole-spine radiographs. PI, which reflects the relationship between the sacral plate and the femoral head, was defined as the angle between a line drawn from the center of the femoral head to the center of the sacral plate and a line perpendicular to the center of the sacral plate. Since pelvic movement is limited, PI can be expressed as the sum of SS and PT [19] (Figure 4A). SS was measured as the angle between the horizontal line and the sacral plate. It serves as a parameter for the horizontal plane position of the pelvis and is an important determinant of lumbar curvature size and shape [20] (Figure 4B). PT was measured as the angle between the vertical line passing through the femoral head center and the line connecting the femoral axis center to the sacral endplate (Figure 4C). PT is a vertical plane parameter that describes the anterior positioning of the pelvis relative to the transverse axis passing through the hip joint [21].

#### 2.3.5. Alignment Parameter of the Lumbosacral Region

To evaluate sagittal alignment in the lumbosacral region, the lumbar lordosis (LL) angle was measured on weight-bearing whole-spine lateral radiographs. LL was defined as the Cobb angle between the superior endplate of the first lumbar vertebra and the inferior endplate of the first sacral vertebra (Figure 4D).

### 2.4. Data Extraction

Parameters were measured using anatomical landmarks on plain radiographs in the Picture Archiving and Communication System (PACS). All angular measurements were performed by a single trained investigator experienced in musculoskeletal radiographic analysis to ensure measurement consistency. Throughout the entire study period, the same radiographic equipment and PACS software (INFINITT Healthcare Co., Ltd., Seoul, Republic of Korea; version unknown) were used without any hardware or software updates. All radiographs were obtained using a standardized digital radiography system under routine clinical imaging protocols for musculoskeletal evaluation. During imaging, participants were instructed to stand in an upright neutral posture with both knees fully extended and arms placed comfortably in front of the body to avoid obstruction of the spine. The radiographic beam was centered at the lumbosacral junction, and efforts were made to obtain true lateral projections of the pelvis and lumbar spine by aligning the patient’s shoulders and pelvis perpendicular to the detector. All measurements were conducted following a standardized protocol established at the beginning of the study to ensure consistency and reproducibility. Each participant’s measurements were performed within a single imaging session to eliminate inter-session variability. Angular parameters, which are less sensitive to scale variations, were measured, and the parameter values were recorded along with their means and standard deviations. The measured values were analyzed by reproducing and reviewing existing studies based on research data, and correlations among parameters related to body alignment and balance centered on the foot joints were verified. Microsoft Excel 2019 (Microsoft Corp, Released 2021, Redmond, WA, USA) was used for data extraction and organization.

### 2.5. Statistical Analysis

SPSS Statistics^®^ (version 29.0, IBM SPSS Statistics, IBM Corp., Armonk, NY, USA) was used for statistical analysis. A *p*-value of less than 0.05 was considered statistically significant. Normality was tested using the Shapiro–Wilk test, and homogeneity of variance was assessed using Levene’s test. All variables satisfied these assumptions; otherwise, the nonparametric Kruskal–Wallis test was applied as an alternative to one-way ANOVA. To minimize the risk of Type I errors resulting from multiple comparisons, the false discovery rate (FDR) correction method was additionally applied.

Participants were classified into three subgroups according to foot structure based on the calcaneal inclination angle (CIA): pes planus (*n* = 5), normal (*n* = 57 for the right foot, 58 for the left foot), and pes cavus (*n* = 38 for the right foot, 37 for the left foot). The overall study population consisted of 36 men and 64 women, and these subgroup sample sizes met the minimum requirements determined by the a priori power analysis described above.

One-way ANOVA (or Kruskal–Wallis test when assumptions were violated) was performed to compare differences among foot structure groups separately for the right and left feet. In addition to *p*-values, effect size metrics (η^2^) were calculated for each ANOVA result to assess the magnitude of group differences and improve the interpretability of the findings.

Correlation analysis was conducted to evaluate the Pearson correlation coefficients between foot and body alignment parameters across all classifications. Although anthropometric variables such as sex, BMI, and limb dominance were collected, multivariate regression models were not applied in the present study because the primary objective was to explore radiographic angular relationships. Future research using multivariate analytical approaches will be required to control for potential confounding variables and to determine whether the observed associations remain significant after adjustment. In interpreting correlation strength, 0.01 ≤ r < 0.30 was defined as weak, 0.30 ≤ r < 0.70 as moderate, and 0.70 ≤ r ≤ 1.00 as strong. Positive and negative coefficient values indicated positive and negative relationships, respectively.

## 3. Results

### 3.1. Demographic Data of Participants

After reviewing the medical records, 100 subjects were selected and included in the study. This number represents all eligible patients who met the predefined inclusion criteria during the study period. The study population consisted of 36 males and 64 females, with a mean age of 32.5 ± 11.3 years. Participants’ vital signs were within normal ranges, with a mean systolic and diastolic blood pressure of 120.6 ± 10.5 mmHg and 75.6 ± 8.5 mmHg, respectively, a mean heart rate of 78.9 ± 10.6 beats per minute, and a mean body temperature of 36.7 ± 0.3 °C. Anthropometric measurements showed a mean height of 165.0 ± 8.2 cm, a mean weight of 62.8 ± 10.8 kg, and a mean body mass index of 22.9 ± 2.7 kg/m^2^ (Table 1).

### 3.2. Analysis of Participants’ CIA Measurements

Analysis of the mean and standard deviation of the CIA measurements revealed that the right foot had a mean value of 24.01° ± 4.82°, while the left foot had a mean value of 24.29° ± 4.75°. When analyzed by subgroups, CIA measurements for the pes planus, normal, and pes cavus groups were 15.06° ± 2.51°, 21.50° ± 2.38°, and 28.97° ± 2.68° for the right foot, and 15.60° ± 1.52°, 21.82° ± 2.08°, and 29.34° ± 2.90° for the left foot, respectively. Based on foot type, the angle differences ranged from 6.22° to 7.52° (Table 2).

### 3.3. Body Alignment Parameter Measurements and ANOVA Analysis of Mean Parameters According to Foot Structure

Due to the small sample size of the pes planus subgroup (*n* = 5), results related to this group are presented descriptively and should be interpreted as preliminary observations rather than evidence of group-level differences.

Analysis of the ankle parameters showed mean TTA values of 2.01° (right) and 1.56° (left). Although both sides demonstrated an increasing trend in TTA values from the pes planus to the pes cavus groups (right: 1.47°, 1.84°, and 2.34°; left: 1.45°, 1.69°, and 2.60°), these differences were not statistically significant. Similar trends were observed in TA measurements, with overall means of 91.68° (right) and 91.50° (left), showing slight increases across foot types, but without statistical significance.

Knee alignment analysis revealed mean Q-angle values of 10.08° (right) and 10.35° (left). The right side showed a progressive increase in Q-angle values from the pes cavus to the pes planus groups (9.68°, 10.19°, and 11.87°, respectively). In contrast, the left side demonstrated notably different measurements across groups (10.80°, 10.55°, and 10.87°), with the pes planus group showing the lowest value.

The pelvic parameters showed consistent patterns across measurements. The mean PI (53.79°) demonstrated a decreasing trend from the pes planus to the pes cavus group in both feet. The PT and SS measurements followed similar patterns, with overall means of 13.18° and 40.61°, respectively. LL measurements (mean, 58.02°) also showed a gradual decrease from pes planus to pes cavus, although none of these trends were statistically significant (Table 3). The correlation analysis results are presented in Table 4.

## 4. Discussion

Excessive adduction of the midfoot can induce changes in tibial and femoral rotation, altering the normal dynamic control of these joints, and potentially causing a cascading effect through the ankle, knee, and pelvis [4]. These changes can lead to instability and misalignment of the knees and pelvis. In other words, the foot structure can cause a series of musculoskeletal problems, including those of the pelvis, while knee joint issues can also affect the pelvic and spinal joints, potentially resulting in back pain or abnormal gait [4]. More specifically, pes planus (flat feet) can impair proprioception in the sole, negatively affecting lower limb muscle activity and potentially disrupting the hip joint range of motion and knee joint alignment [22]. Additionally, pes cavus leads to instability due to decreased ankle adduction and abduction ability, which sequentially increases the burden on the leg and pelvic alignment and ultimately causes abnormal stress in the lumbar region [23]. Therefore, this study analyzed the correlation between foot parameters and ankle, knee, pelvic, and lumbosacral alignment using plain radiography. Although most group differences were not statistically significant, the directional trends observed across foot morphology groups may reflect biomechanical adaptations along the lower-limb kinetic chain. Overall, the present findings suggest that variations in foot arch height, particularly in pes cavus, may be associated with measurable changes in knee and spinopelvic alignment parameters within the ascending kinetic chain.

Given the imbalance in subgroup sample sizes, statistical significance was not the primary focus of the subgroup analyses. Instead, the observed direction and magnitude of effects were considered to explore potential biomechanical patterns for hypothesis generation.

These moderate negative correlations between foot and pelvic parameters suggest that alterations in foot arch height may influence the sagittal balance of the pelvis and lumbar spine. Increased or decreased arch height can change the load distribution across the lower limbs, resulting in compensatory pelvic tilt and lumbar adjustments to maintain postural equilibrium. Such compensations may increase stress on the sacroiliac joint and lumbar intervertebral discs, contributing to musculoskeletal discomfort and low back pain. Accordingly, these radiographic relationships may reflect underlying functional impairments in postural control rather than isolated structural variations. This interpretation aligns with previous epidemiological findings linking abnormal foot posture to lumbopelvic disorders, such as pelvic tilt asymmetry and chronic low back pain.

The statistically significant correlations derived from this study are as follows. First, among knee parameters, a moderate negative correlation (r = −0.3623) was found between the CIA and ipsilateral Q-angle in the left pes cavus group. These results provide important insights into the anatomical and physiological characteristics of pes cavus and its impact on foot and knee alignment. When the foot arch increases in height, the medial part of the foot becomes more distant from the ground, and the lateral part bears more weight. This lateral rotation of the foot induces lateral tibial rotation, leading to changes in tibial and femoral alignment. The Q-angle, defined as the angle between the femur and tibia based on the lines connecting the patella with the femur and the tibial tubercle, decreases when the tibia rotates laterally due to pes cavus [24]. This negative correlation between increased CIA and the Q-angle was clearly demonstrated in our results, emphasizing the impact of pes cavus on knee alignment and function, and providing important information for evaluating knee stability-related issues.

Second, correlation analysis demonstrated a moderate negative correlation (r = −0.5677) between the CIA and PI in the right pes cavus group. This finding suggests that an increased foot arch height caused by pes cavus may lead to lateral rotation of both the foot and tibia, subsequently influencing pelvic tilt. A similar tendency was observed as a moderate negative correlation (r = −0.3587) between CIA and SS in the left normal group. Abnormal pelvic alignment can impose stress on the sacroiliac joint, potentially resulting in pain, dysfunction, and gait problems [25], while also compromising the function of the peripelvic soft tissues [26]. Furthermore, posterior pelvic tilt increases contact between the femoral head and acetabulum, thereby elevating the risk of impingement syndrome. These findings suggest that foot arch height and pelvic tilt may be biomechanically related in individuals with pes cavus. However, given the retrospective and correlational nature of this study, these observations should be interpreted as hypothesis-generating rather than direct clinical recommendations.

Third, among the lumbosacral parameters, moderate negative correlations were observed between the CIA and LL in both the right (r = −0.5677) and left (r = −0.3355) pes cavus groups. This trend aligns with the pelvic findings: as the foot arch heightens, the pelvis tends to tilt posteriorly, which may in turn reduce LL [27]. Additionally, PI, which reflects the relative position of the sacrum and pelvis, is strongly associated with spinal inclination, necessitating a comprehensive evaluation of the lumbosacrum with the pelvis when assessing foot and body alignment. Decreased LL can increase disc pressure, leading to back pain and a higher risk of herniation [28]. Moreover, abnormal spinal alignment can compromise balance and gait ability, negatively impacting overall function [29]. Therefore, pelvic and foot alignment should be examined simultaneously for accurate diagnosis and targeted treatment.

This study retrospectively analyzed patient charts to identify ascending patterns associated with morphological abnormalities of the foot. CMM recognizes various factors that affect body alignment, including descending, ascending, and pelvic girdle patterns [1]. However, it emphasizes the ascending pattern and generally proceeds with treatment from the bottom to the top [30]. The CMM explains the mechanism of the ascending pattern as follows: foot hyperpronation induces internal rotation of the tibia and femur, resulting in decreased femoral head height and contralateral rotation of the L5 vertebral body. Additionally, femoral internal rotation causes lordotic deformation of the lumbar spine [31]. Although lumbar and pelvic deformations can affect superior structures, the patient may also have a pelvic girdle pattern or descending pattern. Nevertheless, due to limitations in patient records, establishing causality was difficult; therefore, the body parameters selected in this study were restricted to regions below the lumbosacral region.

In addition to the CMM perspective, the observed ascending mechanism can be interpreted within conventional biomechanical frameworks. From a kinetic chain standpoint, misalignment in the foot and ankle induces compensatory adjustments through the tibia, femur, and pelvis, ultimately affecting sagittal spinal balance. Such compensations are part of an integrated neuromusculoskeletal response to maintain equilibrium and minimize energy expenditure. Modern biomechanical studies also support this interconnectedness, demonstrating that alterations in lower-limb alignment can modify pelvic tilt, sacral slope, and lumbar lordosis, thereby influencing overall postural stability. Integrating these principles enhances the global relevance of the current findings by bridging traditional CMM theory with contemporary biomechanical understanding. Within this biomechanical framework, these structural adaptations provide a meaningful basis for understanding the clinical manifestations of postural imbalance, linking distal foot morphology to functional and symptomatic changes in proximal musculoskeletal regions.

Radiological parameters of the foot include various angle measurement methods based on the talar, first metatarsal, and calcaneal axes. For example, the talonavicular coverage, talus-second metatarsal, and calcaneal-first metatarsal angles reflect the alignment status in specific parts or planes of the foot [32,33]. Accordingly, this study should be regarded as a hypothesis-generating investigation, providing foundational data for future adequately powered confirmatory studies. However, this study measured the CIA, a foot angle indicator widely used in clinical research owing to its high reliability, reproducibility, and inter-rater consistency. In actual clinical practice, the most reflective indicator of the foot arch form is the normalized navicular height, which shows a strong correlation with the CIA, a lateral view angle, providing the most comprehensive and useful information for evaluating the overall biomechanical state of the foot [34]. CIA measurements using plain radiography represent the calcaneal angle and evaluate the posterior structure of the foot. In pes planus, instability of the arch can occur, with excessive pressure on the plantar surface, especially in the central supporting area of the arch, which is typically indicated by the CIA being lower than the normal range [35]. Additionally, arch collapse in pes planus can increase adduction toward the first metatarsal, potentially causing additional pressure [36].

Generally, flat feet are more common than pes cavus feet, particularly in children and older adults [37]. However, in the present study, the ratio of pes planus to pes cavus was 5:38 and 5:37 for the right and left feet, respectively, indicating a higher proportion of pes cavus. This was primarily due to the study’s mean participant age of 32.5 years, excluding children and older adults. Additionally, the CIA measurement criteria for defining pes planus and pes cavus can vary among studies, and this study may have used criteria that are more sensitive in detecting pes cavus than pes planus. Although some studies set the pes cavus criterion at 30° CIA [13], this study defined it as 25°. Although pes cavus is clinically less common, medical issues related to pes cavus can be serious, including ankle instability and long-term degenerative changes in the ankle joint [38], and pes cavus itself may be a symptom of neurological conditions [39], making it important to detect and manage these issues accurately and early.

Based on the results of this study, when patients with clinically observed pes planus or pes cavus complain of musculoskeletal pain, evaluation and diagnosis should consider the ascending pattern of body alignment. However, when evaluating the feet clinically, it is important to comprehensively assess metatarsal position, foot arch form, ankle and foot stability, and leg muscle balance, in addition to measuring CIA through radiographic imaging [40]. Using plantar pressure analysis, body type assessment, gait analysis, and posture analysis together enables a more precise evaluation [41]. If a comprehensive ascending pattern diagnosis is made, treatment should approach both the problematic area and the foot.

This study had several limitations. First, some radiographic images showed variations in contrast and clarity, which may have influenced the identification of anatomical landmarks during angle measurements. Second, a key limitation of this study is the highly imbalanced distribution of foot morphology subgroups, particularly the small number of participants in the pes planus group. In addition, the study population included a higher proportion of female participants. Furthermore, body mass index (BMI) may influence spinopelvic alignment and lower-limb biomechanics. Although BMI data were collected in this study, its potential effect on alignment parameters was not specifically analyzed, which represents another limitation of the current analysis. Since pelvic and knee alignment parameters may differ according to sex-related anatomical characteristics, this imbalance may have influenced the observed correlations. Therefore, future studies with sex-balanced samples will be required to further investigate these effects. Therefore, findings related to these subgroups should be interpreted with caution and cannot be generalized. Third, additional measurable parameters from the AP foot radiographs were not measured. Additionally, sagittal alignment parameters of the knee, such as knee flexion and extension angles, were not evaluated in this study. Because knee sagittal alignment may influence pelvic tilt and lumbar lordosis through the kinetic chain, future studies should include these parameters to provide a more comprehensive analysis of lower-limb biomechanics. Since changes in both the AP and lateral aspects of the foot can affect the lower extremities [42], a more complex analysis, including foot AP imaging, is needed to verify the correlations between foot parameters and body alignment parameters. Fourth, correlations among body alignment parameters excluding the foot were not assessed. Since correlation does not imply causation, longitudinal studies are needed to clarify the causal relationships between foot and body parameters. Finally, to strengthen the clinical applicability of these findings, follow-up observations should track corrective effects through radiological before-and-after comparisons within the same subject group by measuring foot pronation angle and body alignment parameters before and after correcting foot structure and arch abnormalities using insoles. Ideally, radiographic examination should be performed with legs parallel and spread hip-width apart; however, in this study, images were taken with the feet together. This may have affected the correlation analysis; therefore, this point should be considered in future research.

However, this study was significant in that it observed correlations between the ankle, knee, pelvis, and lumbosacral parameters that constitute the body’s gravity line, centered on foot parameters, and verified the ascending pattern of body misalignment in CMM using radiological data. Currently, CMM continues to diagnose displacement through medical imaging [43] or with artificial intelligence-assisted analysis of imaging results [7]. In CMM, which emphasizes the ascending pattern, the results of this study provide a starting point for a more precise evaluation of the foot structure’s impact on the entire musculoskeletal system and an opportunity to enhance CMM’s diagnostic precision by identifying the root cause of spinal malposition.

In this study, radiographs were obtained with the participants’ feet positioned together. This stance may have slightly altered pelvic and spinal alignment parameters, including PI, PT, and LL, due to restricted lower-limb base width. Such positioning could increase pelvic retroversion and decrease lumbar lordosis compared with a neutral stance. Therefore, future studies should employ standardized radiographic acquisition with feet placed parallel and hip-width apart to minimize these potential measurement biases. Nevertheless, the present results remain meaningful because the same imaging posture was consistently applied to all participants under identical conditions throughout the study.

Furthermore, the retrospective nature and relatively modest sample size may limit the statistical power of this study. Nevertheless, to reduce the risk of false positive findings arising from multiple comparisons, a false discovery rate (FDR) correction and effect size (η^2^) metrics were applied as described in the Section 2. These approaches helped enhance the interpretability and practical relevance of the results, although future studies with larger, prospectively powered samples are recommended to validate these findings.

In addition, although anthropometric variables such as BMI, sex, and limb dominance were collected, they were not incorporated into the current correlation analysis to maintain the focus on radiographic angular relationships. Future research employing multivariate or regression-based approaches will be needed to control for these covariates and to refine the understanding of their potential effects on spinopelvic and lower-limb parameters.

Future studies should evaluate the relationship between foot structure and body alignment using large-scale samples across various age groups and activity levels. Such research is important for the detailed analysis of sex- and age-specific differences in foot structure and body alignment and for developing personalized assessment and treatment strategies. It is essential to evaluate the clinical effects of treatment methods that consider foot structure, and measuring the CIA is important as a key indicator for diagnosing foot arch abnormalities and establishing appropriate correction plans. Through such research, more effective evaluation and treatment methods can be developed through a deeper understanding of the interaction between structural foot changes and body alignment, thereby contributing to foot health promotion.

## 5. Conclusions

This study analyzed how structural changes in the foot influence alignment in other parts of the body. The results revealed negative correlations between the CIA and the Q-angle, PI, SS, and LL. These findings provide important baseline data for understanding the potential relationship between foot structural changes and alignment of the ankle, knee, pelvis, and lumbosacral region. However, these observations should be interpreted as preliminary radiographic evidence and require confirmation through larger prospective and interventional studies. From a clinical perspective, the observed intersegmental correlations support the inclusion of foot morphology assessment in early screening protocols and in the planning of rehabilitation programs for patients with postural imbalance. This study represents Level III evidence, based on a retrospective observational design, and provides a foundation for future prospective and interventional research.

## Figures and Tables

**Table 1:** Demographic characteristics of participants.

Variable		(*n* = 100)
Sex	Male (*n*)	36
	Female (*n*)	64
Age (years; mean [SD])		32.5 (11.3)
Blood pressure	Systolic (mmHg; mean [SD])	120.6 (10.5)
	Diastolic (mmHg; mean [SD])	75.6 (8.5)
Heart rate (bpm; mean [SD])		78.9 (10.6)
Body temperature (°C; mean [SD])		36.7 (0.3)
Height (cm; mean [SD])		165.0 (8.2)
Weight (kg; mean [SD])		62.8 (10.8)
Body mass index (kg/m^2^; mean [SD])		22.9 (2.7)

SD: standard deviation.

**Table 2:** Analysis of the parameters of the foot.

Variable	N	Mean ± SD (°)	Sub-Variable	N	Mean ± SD (°)
CIA (Rt.)	100	24.01 ± 4.81	Pes planus	5	15.06 ± 2.51
			Normal	57	21.50 ± 2.38
			Pes cavus	38	28.97 ± 2.68
CIA (Lt.)	100	24.29 ± 4.75	Pes planus	5	15.60 ± 1.52
			Normal	58	21.82 ± 2.08
			Pes cavus	37	29.34 ± 2.90

CIA: Calcaneal Inclination Angle; SD: Standard Deviation; All angular values are expressed in degrees (°).

**Table 3:** Mean value and ANOVA analysis of body alignment parameters according to the foot type.

Variable	Sub-Variable	N	Mean ± SD (°)	F	*p*-Value	Variable	Sub-Variable	N	Mean ± SD (°)	F	*p*-Value
TTA—Rt. foot	All	100	2.01 ± 3.09	0.37	>0.05	TTA—Lt. foot	All	100	1.56 ± 1.35	1.09	>0.05
	Pes planus	5	1.47 ± 0.87				Pes planus	5	1.45 ± 0.93		
	Normal	57	1.84 ± 1.34				Normal	58	1.69 ± 1.15		
	Pes cavus	38	2.34 ± 4.74				Pes cavus	37	2.60 ± 4.84		
TA—Rt. foot	All	100	91.68 ± 3.69	0.41	>0.05	TA—Lt. foot	All	100	91.50 ± 3.64	0.79	>0.05
	Pes planus	5	90.88 ± 2.10				Pes planus	5	91.32 ± 0.65		
	Normal	57	91.29 ± 0.49				Normal	58	91.32 ± 0.55		
	Pes cavus	38	91.91 ± 0.55				Pes cavus	37	92.29 ± 0.50		
Q-angle—Rt. foot	All	100	10.08 ± 6.29	0.28	>0.05	Q-angle—Lt. foot	All	100	10.35 ± 6.46	2.01	>0.05
	Pes planus	5	11.87 ± 10.08				Pes planus	5	10.87 ± 5.00		
	Normal	57	10.19 ± 6.10				Normal	58	10.55 ± 5.24		
	Pes cavus	38	9.68 ± 6.18				Pes cavus	37	10.80 ± 6.79		
PI—Rt. foot	All	100	53.79 ± 14.68	2.09	>0.05	PI—Lt. foot	All	100	53.79 ± 14.68	0.61	>0.05
	Pes planus	5	56.66 ± 8.81				Pes planus	5	60.52 ± 10.19		
	Normal	57	56.07 ± 15.10				Normal	58	53.87 ± 14.90		
	Pes cavus	38	50.00 ± 14.13				Pes cavus	37	52.77 ± 14.92		
PT—Rt. foot	All	100	13.18 ± 8.02	3.30	0.041	PT—Lt. foot	All	100	13.18 ± 8.02	0.36	>0.05
	Pes planus	5	12.70 ± 1.43				Pes planus	5	15.99 ± 1.43		
	Normal	57	14.90 ± 8.79				Normal	58	13.24 ± 1.12		
	Pes cavus	38	10.68 ± 6.62				Pes cavus	37	12.73 ± 1.26		
SS—Rt. foot	All	100	40.61 ± 10.82	0.58	>0.05	SS—Lt. foot	All	100	40.61 ± 10.82	0.37	>0.05
	Pes planus	5	43.95 ± 8.77				Pes planus	5	44.53 ± 9.57		
	Normal	57	41.18 ± 11.21				Normal	58	40.63 ± 11.08		
	Pes cavus	38	39.32 ± 39.32				Pes cavus	37	40.05 ± 10.73		
LL—Rt. foot	All	100	58.02 ± 10.78	0.19	>0.05	LL—Lt. foot	All	100	58.02 ± 10.78	0.23	>0.05
	Pes planus	5	59.20 ± 9.46				Pes planus	5	60.64 ± 9.21		
	Normal	57	58.47 ± 10.81				Normal	58	58.21 ± 10.99		
	Pes cavus	38	57.18 ± 11.10				Pes cavus	37	57.35 ± 10.85		

LL: lordosis angle between the first lumbar spine and first sacral spine; Lt.: left; PI: pelvic incidence; PT: pelvic tilt; Q-angle: quadriceps angle; Rt.: right; SD: standard deviation; SS: sacral slope; TA: tibial angle; TTA: tibiotalar tilt angle. All angular values are expressed in degrees (°).

**Table 4:** Correlation analysis of body alignment parameters and CIA values.

Variable	Sub-Variable	N	Pearson r	*p*-Value	Variable	Sub-Variable	N	Pearson r	*p*-Value
TTA—Rt. foot	Pes planus	5	0.4135	>0.05	TTA—Lt. foot	Pes planus	5	−0.1639	>0.05
	Normal	57	0.1760	>0.05		Normal	58	−0.1376	>0.05
	Pes cavus	38	−0.1525	>0.05		Pes cavus	37	−0.0476	>0.05
TA—Rt. foot	Pes planus	5	−0.0291	>0.05	TA—Lt. foot	Pes planus	5	−0.3247	>0.05
	Normal	57	−0.0519	>0.05		Normal	58	−0.0939	>0.05
	Pes cavus	38	0.2218	>0.05		Pes cavus	37	0.0711	>0.05
Q-angle—Rt. foot	Pes planus	5	0.2611	>0.05	Q-angle—Lt. foot	Pes planus	5	−0.1630	>0.05
	Normal	57	−0.0474	>0.05		Normal	58	−0.1088	>0.05
	Pes cavus	38	−0.0831	>0.05		Pes cavus	37	−0.3623	0.0275
PI—Rt. Foot	Pes planus	5	−0.6009	>0.05	PI—Lt. foot	Pes planus	5	−0.5802	>0.05
	Normal	57	−0.1758	>0.05		Normal	58	−0.3308	0.0112
	Pes cavus	38	−0.5677	0.0002		Pes cavus	37	−0.1048	>0.05
PT—Rt. foot	Pes planus	5	0.15180	>0.05	PT—Lt. foot	Pes planus	5	0.4326	>0.05
	Normal	57	−0.05155	>0.05		Normal	58	−0.1120	>0.05
	Pes cavus	38	0.13980	>0.05		Pes cavus	37	−0.0452	>0.05
SS—Rt. foot	Pes planus	5	−0.6289	>0.05	SS—Lt. foot	Pes planus	5	−0.7628	>0.05
	Normal	57	−0.1962	>0.05		Normal	58	−0.3587	0.0057
	Pes cavus	38	−0.1255	>0.05		Pes cavus	37	−0.1134	>0.05
LL—Rt. foot	Pes planus	5	−0.02871	>0.05	LL—Lt. foot	Pes planus	5	−0.1631	>0.05
	Normal	57	−0.09353	>0.05		Normal	58	−0.1691	>0.05
	Pes cavus	38	−0.56770	0.0002		Pes cavus	37	−0.3355	0.0423

CIA: calcaneal inclination angle; LL: lordosis angle between the first lumbar spine and first sacral spine; Lt.: left; PI: pelvic incidence; PT: pelvic tilt; Q-angle: quadriceps angle; Rt.: right; SS: sacral slope; TA: tibial angle; TTA: tibiotalar tilt angle.

## Data Availability

The data presented in this study are available from the corresponding author upon reasonable request.

## Abbreviations

The following abbreviations are used in this manuscript:

CMM	Chuna Manual Medicine
CIA	Calcaneal Inclination Angle
TTA	Tibiotalar Tilt Angle
TA	Tibiotalar Angle
Q-angle	Quadriceps Angle
PI	Pelvic Incidence
PT	Pelvic Tilt
SS	Sacral Slope
LL	L1–S1 Lordosis
AP	Anteroposterior
PACS	Picture Archiving and Communication System
SD	Standard Deviation
ANOVA	Analysis of Variance
